# Effect of NMSO3 treatment in a murine model of human metapneumovirus infection

**DOI:** 10.1099/vir.0.2008/003301-0

**Published:** 2008-11

**Authors:** Leanne Spetch, Terry L. Bowlin, Antonella Casola

**Affiliations:** 1 Department of Pediatrics, University of Texas Medical Branch, Galveston, TX, USA; 2 Microbiotix Inc., Worcester, MA, USA; 3 Department of Microbiology and Immunology, University of Texas Medical Branch, Galveston, TX, USA; 4 Sealy Center for Vaccine Development, University of Texas Medical Branch, Galveston, TX, USA

## Abstract

BALB/c mice infected with human metapneumovirus (hMPV) were treated with the sulfated sialyl lipid NMSO3 (one dose of 50 mg kg^−1^) given at the time of infection. NMSO3 significantly reduced viral replication in the lungs, as well as hMPV-induced body weight loss, pulmonary inflammation and cytokine production, suggesting that antiviral treatment initiated at the beginning of viral infection can modify hMPV-induced disease.

Human metapneumovirus (hMPV) is a recently identified human respiratory viral pathogen belonging to the family *Paramyxoviridae* (van den Hoogen *et al.*, 2001). hMPV is associated with upper and lower respiratory tract infections in children and adults, as reported in studies worldwide. According to various reports, between 5 and 12 % of all respiratory tract infections in younger infants are caused by hMPV, second only to respiratory syncytial virus (RSV), and it may account for 10 % of all hospitalizations of elderly patients with respiratory tract infections (reviewed by Hamelin & Boivin, 2005). No specific antivirals or other therapeutic agents are licensed to treat hMPV infection. Similarly, no vaccine is currently available. We and others have recently identified BALB/c mice as an effective murine model to investigate hMPV-induced airway disease (Alvarez *et al.*, 2004; Guerrero-Plata *et al.*, 2005; Hamelin *et al.*, 2005). Ribavirin, a nucleoside analogue of guanosine used in the treatment of RSV infection, has recently been shown to affect hMPV replication *in vitro* and *in vivo* (Hamelin *et al.*, 2006; Wyde *et al.*, 2003). Similarly, NMSO3, a sulfated sialyl lipid with potent antiviral activity against RSV, has recently been shown to inhibit hMPV replication *in vitro* (Wyde *et al.*, 2004). In this study, we assessed the effect of NMSO3 treatment on hMPV replication and pulmonary inflammation *in vivo*, using a BALB/c model of infection.

Female BALB/c mice (Harlan Sprague Dawley), 6–7 weeks old, were housed under pathogen-free conditions. A total of four experimental groups consisting of two treatment groups (carrier and NMSO3) for each infection group (mock- and hMPV-infected) was used for all experiments. For each experiment, four to five mice were used per group. Experiments were repeated two to three times. There was no effect of the vehicle by itself; therefore, the groups of mock-infected+carrier and hMPV-infected+carrier are identified in the text and figures as mock- and hMPV-infected. Under light anaesthesia, mice were inoculated intranasally (i.n.) with 1×10^7^ TCID_50_ hMPV in a total volume of 50 μl. This volume of inoculum has been shown to deliver the virus directly to the lungs. At 30–60 min after viral inoculation, mice were administered NMSO3 i.n. at a concentration of 10, 50 or 100 mg kg^−1^ in a total volume of 50 μl. NMSO3 was prepared in water at a stock concentration of 100 mg ml^−1^ and then diluted in PBS to the final desired concentration immediately before administration. Mice were weighed daily from day 0 to 10 post-infection (p.i.). At days 1 and 4 p.i., mice were sacrificed to determine lung viral titres and cellular inflammation, and to measure cytokine and chemokine production, as described previously (Guerrero-Plata *et al.*, 2005, 2006; Haeberle *et al.*, 2001).

To determine the effects of NMSO3 on hMPV infection, we initially examined whether NMSO3 altered hMPV replication *in vivo*. We tested three different concentrations of NMSO3, given on the first day of hMPV infection, based on a previous study that used NMSO3 in a rat model of RSV infection, showing amelioration of RSV-induced clinical illness (Kimura *et al.*, 2000). In our mouse model of hMPV infection, increased viral titres could be detected as early as day 3 p.i., with peak viral titres occurring at day 5 p.i. and viral clearance by day 7 p.i. (data not shown). NMSO3 administration of 10 mg kg^−1^ only marginally reduced hMPV replication from 4.5±0.1 to 4.1±0.05 log_10_, whilst treatment with 50 mg kg^−1^ significantly decreased peak viral load by about 1 log, from 5.12±0.31 to 4.1±0.32 log_10_. Administration of 100 mg kg^−1^ resulted in a further reduction in viral replication (5.2±0.1 to 3.9±0.2 log_10_), but was also associated with toxicity, as indicated by an increased loss of body weight, an important parameter of hMPV-induced clinical illness (see below), in the animals infected and treated with NMSO3 compared with the animals infected with virus alone.

Mice infected with 1×10^7^ TCID_50_ hMPV lost 5–10 % of their original body weight, with a peak that occurred between days 1 and 2 p.i. They gradually returned to their original weight and then started to lose weight again for an additional 4–5 days before returning to baseline levels. Mock-infected mice did not loose weight (data not shown). HMPV-infected mice treated with NMSO3 showed a similar body weight loss in the first 3 days of infection; however, they did not loose additional weight after returning to baseline levels, as shown in Fig. 1(a).

We then tested the efficacy of 50 mg NMSO3 kg^−1^ given at 1 day p.i. NMSO3 treatment was still effective in reducing viral replication, although not as well as administration immediately after infection (reduction of ∼0.6 log vs 1 log in viral titres). Mice infected with hMPV and treated with NMSO3 the day after infection showed a body weight loss similar to the untreated infected mice, but seemed to recover faster than the untreated mice, as shown in Fig. 1(b). As administering 50 mg NMSO3 kg^−1^ at the time of infection seemed to be the most effective treatment in modulating hMPV-induced clinical disease, all subsequent experiments were performed using this treatment protocol.

To determine whether NMSO3 altered hMPV-induced lung inflammation, total and differential cell counts in bronchoalveolar lavages (BALs) were measured. In mock-infected mice, total lung cell count was between 1×10^4^ and 1.5×10^4^, with macrophages representing the majority of the cell population. We observed a significant attenuation of total cellular influx following NMSO3 treatment in HMPV-infected mice on day 1 p.i. (*P*=0.056), with a similar trend at day 4 p.i. (Fig. 2a[Fig f2]). HMPV infection induces a significant recruitment of neutrophils to the lung within the first 3 days of infection, whilst mononuclear cells, including macrophages/monocytes and lymphocytes, start to increase at day 3 p.i., representing the majority of the lung inflammatory cells from day 5 p.i. (Kolli *et al.*, 2008). NMSO3 administration did not cause significant changes in the distribution of the inflammatory cell population at day 1 p.i. between treated and untreated groups, whilst there was a significant increase in neutrophil recruitment in the airways by day 4 p.i. (Fig. 2b[Fig f2]).

HMPV infection of BALB/c mice results in significant production of cytokines and chemokines (Alvarez *et al.*, 2004; Guerrero-Plata *et al.*, 2005; Hamelin *et al.*, 2005), which are likely to play a major role in pulmonary inflammation. Therefore, we determined whether NMSO3 treatment could modulate hMPV-induced cytokine and chemokine secretion by analysing their levels in BAL samples collected at days 1 and 4 p.i. As shown in Fig. 3(a), NMSO3 treatment significantly reduced the level of the chemokine RANTES, a potent chemoattractant of mononuclear cells, while increasing the amount of KC, a chemokine important for neutrophil recruitment. The pro-inflammatory cytokines interleukin (IL)-1*α*, IL-1*β*, IL-6 and tumour necrosis factor alpha were strongly upregulated in the lungs of infected animals at day 1 p.i., and NMSO3 treatment significantly reduced all four cytokine protein levels (Fig. 3b[Fig f3]). These cytokines were no longer detectable in the BALs of infected mice at day 4 p.i. In contrast, granulocyte colony-stimulating factor (G-CSF) and gamma interferon (IFN-*γ*) were secreted in significant amounts at day 4 p.i., and were significantly reduced in hMPV-infected animals treated with NMSO3 (Fig. 3c[Fig f3]).

Our study demonstrates that a single administration of NMSO3, at the time of hMPV infection, is effective in reducing viral replication, clinical illness, pulmonary inflammation and chemokine/cytokine production. The initial *in vitro* study suggested that NMSO3 was most effective when present at the beginning of hMPV replication, possibly affecting viral attachment and internalization (Wyde *et al.*, 2004). For this reason, we decided to treat the mice concurrently with hMPV infection. A single dose of 50 mg NMSO3 kg^−1^ was highly effective in preventing significant body weight loss, suggesting that antiviral treatment can have a significant impact on hMPV-induced disease, as also shown recently by the use of ribavirin in a similar model of hMPV infection (Hamelin *et al.*, 2006), in particular when administered at the beginning of infection. NMSO3 given 24 h after infection resulted in only a marginal improvement in hMPV-induced illness, as assessed by body weight loss. This result was similar to what we have shown previously with antioxidant treatment in the context of RSV infection, which was effective if given before or at the moment of infection, but did not result in significant improvement of clinical disease administered 1 day after infection, when clinical disease and lung inflammation are already present (Castro *et al.*, 2006). This finding suggests that early therapeutic intervention may be necessary to improve clinical outcome in both hMPV and RSV infections. In future studies, it would be important to assess other important parameters of clinical illness such as airway hyper-responsiveness, which has been reported in BALB/c mice infected with hMPV or with other paramyxoviruses such as RSV. Similarly, alternative methods of administration, for example via aerosolization, could be tested for efficacy. HMPV infection of BALB/c mice induces a significant lung inflammatory response, with recruitment of phagocytes and then mononuclear cells to the airways, concomitant with the induction of chemokines and cytokines. The role of the inflammatory response in hMPV-induced clinical illness has not yet been investigated. However, the recent finding of a significant reduction of body weight loss following glucocorticoid treatment (Hamelin *et al.*, 2006) in a similar mouse model of hMPV infection suggests that modulating pulmonary inflammation would be beneficial in hMPV-induced disease. A recent *in vitro* study has shown that NMSO3 reduces RSV-induced chemokine production (Miller *et al.*, 2004). Similarly, our results showed that NMSO3 treatment significantly reduced hMPV-induced chemokine/cytokine production, paralleled by a reduction in pulmonary inflammation. The only exception was secretion of KC, a neutrophil chemoattractant, which explains the relatively higher proportion of these phagocytic cells at day 4 p.i. in the NMSO3-treated group. Although this beneficial effect is probably due to the reduction in hMPV replication, it is possible that, in addition to its antiviral activity, NMSO3 may also possess general anti-inflammatory properties, as recently shown *in vitro* by inhibition of adhesion-dependent leukocyte activation (Shodai *et al.*, 2003). We have recently shown that CD4^+^ T lymphocytes play an important role in modulating hMPV-induced clinical illness and pulmonary inflammation (Kolli *et al.*, 2008). It is possible that NMSO3 treatment modulation of the innate response also modified the adaptive immune response, leading to a beneficial effect on disease that is not due only to the antiviral mechanism. Future studies should address whether NMSO3 modifies recruitment/maturation of dendritic cells and whether there are changes in the phenotype of T cells.

In summary, NMSO3 treatment improved several aspects of hMPV-induced disease and should be considered for further evaluation in other animal models.

## Figures and Tables

**Fig. 1. f1:**
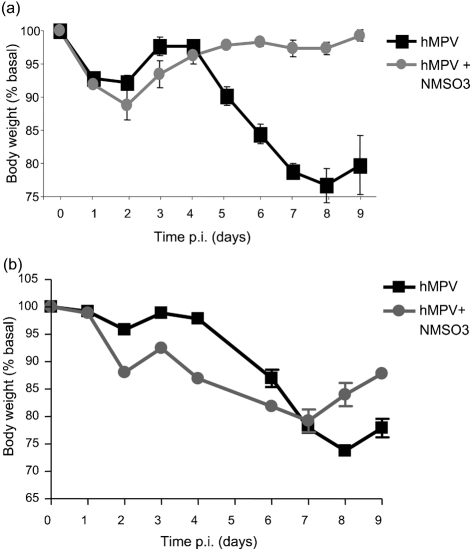
Effect of NMSO3 administration on hMPV-induced body weight loss. Mice were treated with 50 mg NMSO3 kg^−1^ at the time of hMPV infection (a) or at 24 h p.i. (b). The results show a representative diagram from three different experiments. All data points represent the means±sd of four to five animals.

**Fig. 2. f2:**
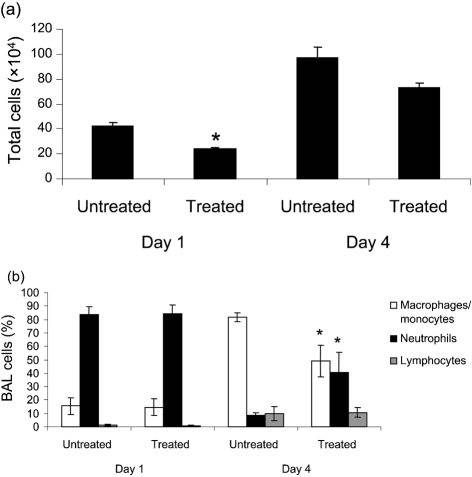
Effect of NMSO3 on airway inflammation. (a) The total number of cells was measured in the BAL of hMPV-infected mice, either untreated or treated with NMSO3, at days 1 and 4 p.i. Data are expressed as means±sd and are representative of three different experiments. (b) Differential cell count was measured in BALs of hMPV-infected mice, untreated or treated with NMSO3, at days 1 and 4 p.i. *, *P*<0.01 relative to hMPV-infected mice.

**Fig. 3. f3:**
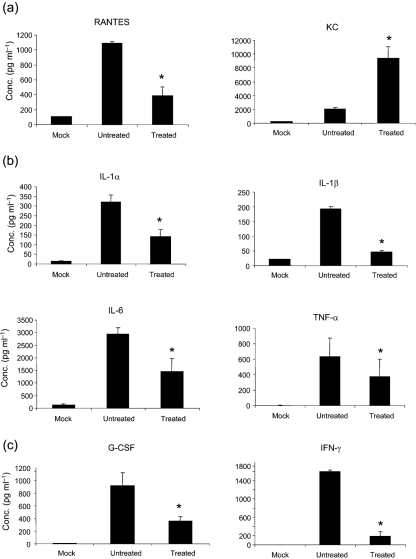
Effect of NMSO3 on cytokine and chemokine release in BAL. Cytokine and chemokine production were measured using a Bioplex array in the BAL of mock- and hMPV-infected mice, either untreated or treated with NMSO3, at days 1 (a, b) and 4 (c) p.i. Data are expressed as means±sd and are representative of three different experiments. *, *P*<0.01 relative to hMPV-infected mice.
